# Assessing Neurocognition (P300) and Correlating Them to Depression Rating Scales in Patients With Major Depressive Disorder

**DOI:** 10.7759/cureus.31084

**Published:** 2022-11-04

**Authors:** Santosh Wakode, Sandip Hulke, Naina S Wakode, Tanusha Pathak, Ragini Shrivastava, Avinash Thakare, Varun Malhotra

**Affiliations:** 1 Physiology, All India Institute of Medical Sciences, Bhopal, IND; 2 Anatomy, Atal Bihari Vajpayee Government Medical College, Vidisha, IND

**Keywords:** psychological disorders, psychology, montgomery-asberg depression rating scale, hamilton rating scale for depression, major depression

## Abstract

Background: Major depression is a chronic condition that may affect cognition. Cognitive disturbances may affect clinical scales used to assess the severity of depression.

Aims: To find an association between cognitive disturbance as objectively recorded using event-related potentials (P300) with the Hamilton rating scale for depression (HAM-D) and Montgomery-Asberg depression rating scale (MADRS) in newly diagnosed cases of major depression.

Methods and material: A cross-sectional study with a sample size of 46 diagnosed cases of major depression. The assessment was done using the HAM-D and MADRS. The P300 assessment was done with the auditory oddball paradigm using the Nihon Kohden NCV-SMG-EP system (Tokyo, Japan).

Statistical analysis: Pearson correlation analysis was used to study the association between various parameters of P300 and the HAM-D and MADRS depression rating scales.

Results: A significant correlation was found between A21- P300 amplitude Cz and the MADRS score. No significant correlation was seen between other P300 parameters and HAM-D and MADRS scales.

Conclusions: As the results were objectively recorded using various parameters of event-related potentials (P300), cognitive impairment was not significantly associated with depression rating scales i.e., the HAM-D and MADRS scores.

## Introduction

Depression is the world's most common cause of disability, impacting nearly 250 million people of all ages [1,2]. Depression can manifest itself in various ways, leading to a clinical diagnosis of major depressive disorder (MDD). Symptoms of MDD include a persistent depressed mood, loss of interest or pleasure, significantly reduced processing speed, working memory, and sustained attention. [1]

The pathophysiological pathways that underpin depression are poorly understood. Neurocognitive impairments, such as episodic memory and executive function deficits, have increased depression severity [3]. Individuals with MDD frequently have impaired processing speed and working memory [1]. These impairments are prevalent depressive symptoms that add to the burden of depression. Deficits in neurocognitive function, in particular, are closely linked to poor psychosocial functioning and poor treatment response [3,4,5].

According to previously published literature, biological, psychological, and social elements all have a role in causing depression which can be defined as the bio-psycho-social model/paradigm. There is a lot of overlap and integration, and the exact causes differ based on the person and situation. Depression is diagnosed solely based on a psychiatric history and a mental state evaluation. Psychological testing might sometimes aid in the diagnosis [2].

Event-related potentials (ERPs), an inexpensive, non-invasive, and reliable method for investigating brain function linked to psychopathology, have been used in psychophysiological research to explore neurocognitive abnormalities in depressive disorders [6]. The P300 is an extensively employed ERP measure to investigate brain activity in depression that is assumed to reflect broad cognitive functions, including attention allocation and working memory [7,8,9]. Research has demonstrated that the P300 has reliable psychometric features in both normative and clinically depressed populations, making it an excellent neural tool for looking into individual differences in neurocognitive functioning in depression [3].

The P300 is a significant positive complex that peaks at 300 milliseconds after the presentation of a stimulus. The P300 travels across the scalp in a centro-parietal pattern, with the most amplitude at location Cz. The P300 ERP is divided into two parts: P3a and P3b. The P3a refers to the early peak with a frontocentral topography, which occurs 20 to 50 ms before the P3b and 250 to 300 ms after the stimulus presentation. It has been found that the P3b is more posterior to the P3a, with a parietal maximal of about 300 to 350 ms. The P3a is thought to direct one's attention to a unique deviant or unfamiliar sound, whereas the P3b represents context updating and memory storage. The P300 is quantified by measuring its amplitude (size) and latency. The voltage difference between a prestimulus baseline and the largest positive-going peak of the ERP waveform within a latency range is defined as amplitude (μV). Latency (ms) is defined as the time between the onset of the stimulus and the point of highest positive amplitude within the latency window {1]. 

Psychological research comparing clinically depressed individuals to healthy individuals, utilizing event-related potentials (ERPs), has consistently demonstrated altered P300 components [6]. Attentional allocation, evaluative processing, context update, and inhibitory control have all been linked to the P300. As a result, the lower P300 amplitude in depression is consistent with the observed attentional and cognitive impairments commonly seen in MDD [1,6].

Several investigations have found that people with current depressive illnesses had lower P300 amplitudes to infrequent target stimuli [10,11]. This P300 effect may be modulated by depression severity [10,12], increased suicidality [13], or specific subtypes of depression such as melancholic subtypes [3].

The oddball task is frequently used as the experimental paradigm in the studies on the P300 in depression discussed above. Participants are expected to count or otherwise respond to one stimulus (the target) while ignoring another stimulus in oddball tasks (i.e., the standard). In oddball activities, the P300's amplitude is quite sensitive to achieving the desired frequency according to target stimuli. As a result, it is feasible that P300 is lower in depression during oddball tasks, reflecting working memory deficits and the extent to which the target frequency is carefully monitored. In addition, the P300 levels that are lower in those who are depressed could indicate a bigger problem in stimulus processing that would be visible in other situations and tasks that require a faster reaction time [3].

Various interview-based tests are also employed for the diagnosis of depression. Some commonly used tests are the Hamilton Rating Scale for Depression (HAM-D) and Montgomery-Asberg Depression Rating Scale (MADRS) [2,14].

The HAM-D scale is regarded as the "gold standard" for determining the severity of depression [2]. Furthermore, this scale is the most sensitive of the regularly used depression scales in identifying changes in the patient's clinical status. Even though the HAM-D has 21 items, the scoring is focused on the first 17. The interview and scoring of the results usually take 15 to 20 minutes. Eight items are rated on a 5-point scale, with 0 indicating no presence and 4 indicating severe presence. Nine objects are graded on a scale of 0 to 2. It offers a straightforward method for determining the intensity of depression; the greater the score, the more severe the depression. The severity of depression was divided into four categories based on the overall HAM-D score: standard (scoring 0-7), mild (8-13), moderate (14-18), severe (19-22), and highly severe depression (score 23) [2]. 

The MADRS was created to measure better depression symptom severity than the commonly used HAM-D for studying antidepressant medication treatment response. Based on their capacity to detect depression change, ten items from the Comprehensive Psychopathological Rating Scale were chosen. The MADRS is now the most widely used clinician-rated measure of depression severity in clinical research, with psychometric qualities equal to or superior to other standardized measures of depression severity [14].

With this study, we attempted to study the association between cognitive disturbance as objectively recorded using event-related potentials (P-300) with the subjective depression rating scale scores (HAM-D scores and MADRS score) in the newly diagnosed case of major depression.

## Materials and methods

Methodology

This s a cross-sectional study done in the Department of Physiology, collaborating with the Psychiatry department. The study includes 46 patients diagnosed with major depression recruited from the psychiatry outpatient department (OPD). The diagnosis was made with a psychiatrist-structured clinical interview for the Diagnostic and Statistical Manual of Mental Disorders, fourth edition, text revision (DSM IV-TR) diagnoses. The study was conducted after the approval of the All India Institute of Medical Sciences (Bhopal, India) Institutional Human Ethics Committee (approval no.:IHEC‑LOP/IM0188)

The inclusion criteria for the study were newly diagnosed patients with major depression. Participants with a known case of seizure disorder, history of alcohol/drug dependence, hearing defect, and antidepressant or psychoactive drugs were excluded from the study. After written informed consent, participants were further assessed with the HAM-D and MADRS by a psychiatrist. Following this, they underwent the P300 test in the physiology department.

Nihon Kohden NCV-SMG-EP system (Tokyo, Japan) was utilized for the P300 assessment. The P300 was recorded in an acoustically and electrically shielded room. The room was dimly lit. The subject was made to sit in a comfortable position or lie down. The procedure was explained to the patient. Then, with all the necessary precautions, the electrodes were placed on the scalp at positions Fz (A11), Cz (A21), and Pz (A31), with the reference electrodes at both the mastoids and Fpz being the ground. The stimulation was provided using an auditory oddball paradigm, the most preferred modality for recording P300 waveforms, wherein the improbability of the task is the main advantage for eliciting the waveform. The subject was given a button that they were supposed to press upon the discrimination of the odd stimulus.

**Auditory oddball paradigm

The oddball auditory paradigm includes a train of auditory stimuli with a specific frequency and intensity interspersed among the rare stimuli with a different intensity. The rare stimuli appear randomly so that the predictability of the appearance of the rare stimuli is ruled out, and the subject has to be attentive to the task at hand. Another advantage of randomizing the rare inspirations is that it rules out the contingency in the thought process that appears with the appearance of the rare stimulus at regular intervals. The oddball paradigm also holds for the omission of a frequent stimulus from the train of stimuli if that is to be identified as a rare stimulus and can also produce the P300 wave.

Statistical analysis

Statistical analysis was done using the statistical software Systac13. Pearson correlation analysis was done to study the association between various parameters of P300 and the HAM-D and MADRS.

## Results

The study was done on 46 patients (mean age 31.04 yrs ± 12.36) diagnosed with major depression.

Table 1 shows the P300 latencies and amplitude Fz (A11), Cz (A21), and Pz levels (A31), as well as the HAM-D, and MADRS scores. No significant correlation was found.

**Table 1:** The P300 latencies; amplitude Fz (A11), Cz (A21), and Pz levels (A31); the HAM-D and MADRS scores. SD: Standard deviation, HAM-D: Hamilton Rating Scale for Depression, MADRS: Montgomery-Asberg Depression Rating Scale

Parameters	Mean ± S.D.
A11- P300 latency Fz (ms)	363.5 ± 73.62
A21- P300 latency Cz (ms)	364.9 ± 73.52
A31- P300 latency Pz (ms)	374.1 ± 71.9
A11- P300 amplitude Fz (µV)	14.1 ± 11.3
A21- P300 amplitude Cz (µV)	13.14 ± 13.9
A31- P300 amplitude Pz (µV)	11.02 ± 5.73
HAM-D score	13.91 ± 6.6
MADRS score	12.91 ± 8.8

The correlation between P300 latencies and HAM-D score is illustrated in Table 2. As is evident from the table, no significant correlation was found.

**Table 2:** Correlation between HAM-D score and P300 latencies HAM-D: Hamilton rating scale for depression

P300 latencies parameters	r-value (Pearson coefficient)	p-value
A11- P300 latency Fz (ms)	0.116	0.443
A21- P300 latency Cz (ms)	0.12	0.42
A31- P300 latency Pz (ms)	0.118	0.43

Table 3 lists the correlation between the HAM-D score and the P300 amplitude. No significant correlation was found.

**Table 3:** Correlation between the HAM-D score and P300 amplitude HAM-D: Hamilton rating scale for depression

P300 latencies parameters	r-value (Pearson coefficient)	p-value
A11- P300 amplitude Fz (µV)	0.083	0.584
A21- P300 amplitude Cz (µV)	0.19	0.2
A31- P300 amplitude Pz (µV)	-0.17	0.25

Table 4 consists of the correlation between the MADRS score and P300 latencies; no significant correlation was found in this case.

**Table 4:** Table 4: Correlation between the MADRS score and P300 latencies MADRS: Montgomery-Asberg depression rating scale

P300 latencies parameters	r-value (Pearson coefficient)	p-value
A11- P300 latency Fz (ms)	-0.041	0.786
A21- P300 latency Cz (ms)	-0.02	0.88
A31- P300 latency Pz (ms)	0.009	0.95

Table 5 illustrates the correlation between the MADRS score and P300 amplitude. A significant correlation was found between the A21- P300 amplitude Cz and the MADRS score. No significant correlation was seen between other P300 parameters and the HAM-D and MADRS scores.

**Table 5:** Correlation between the MADRS score and P300 amplitude *p<0.05 = significant MADRS: Montgomery-Asberg depression rating scale

P300 latencies parameters	r-value (Pearson coefficient)	p-value
A11- P300 amplitude Fz (µV)	0.037	0.8
A21- P300 amplitude Cz (µV)	0.31	0.036*
A31- P300 amplitude Pz (µV)	-0.16	0.28

## Discussion

The study aimed to see if cognitive disruption, as measured by P-300 ERP, could be linked to the HAM-D and MADRS in newly diagnosed cases of major depression. The outcomes of this investigation revealed no significant link between these scales and any of the P300 parameters except the A21-P300 amplitude Cz where only a weak significant correlation was found between P300 amplitude and MADRS.

We also reported prolonged latency and decreased P300 amplitude in depressed patients, which is in alignment with a plethora of previously published literature. Kaustio et al. conducted a comparative study to compare the P300 of depressed patients and healthy controls and could not establish any significant correlation between the two. However, psychotic symptoms are linked to a decrease in amplitude and a longer P300 delay [15]. Similar studies were done by Tripathi et al., who reported prolonged or delayed P-300 latency in the patients of MDD as compared to normal healthy controls. Similar results were observed by Kalayam et al. and Himani et al. [16,17].

A recent study published by Sanpetro et al. reported that P300 amplitude was lower in a flanker task among a large group of clinically depressed adults compared to healthy controls who had never been depressed [3]. Similar results were reported by Klawohn et al. [18]. These findings support the theory that P300 amplitude is a neural correlate of general cognitive processing of target stimuli (i.e., attentiveness, salience allocation) and that decreases in this neural response may represent broader cognitive problems associated with depression [19].

We had come across various studies where P300 latencies were assessed in the Indian population. Singh et al. had latency in mild, moderate, and severe cases of depression as 359.8±53.1, 367.3±53.0, and 432.6±42.6, respectively [20]. Himani et al. [17] found latencies to be 360.4±38.81. Tripathi et al. got a latency of 346.91± 19.515, which is significantly delayed than non-depressed patients. Latency was prolonged in severe cases of depression [2]. In the present study, P300 latencies (ms) at Fz (A11), Cz (A21), and Pz levels (A31) were 363.5 ± 73.62, 364.9 ± 73.52, and 374.1 ± 71.9, respectively. Thus, patients in the present study had shown cognitive impairment.

A cognitive deficit is expected in major depression patients. It may be associated with the inability to pay attention, process information quickly, remember and recall information, respond to information quickly, think critically, plan, organize, solve problems, and initiate speech [21,22]. The patient may have difficulty thinking and concentrating, and making decisions.

Depression patients are monitored with various scales. In the present study, the scales used were HAM-D and MADRS. We observed a poor correlation between P300 ERP parameters and these depression rating scale scores. Thus, cognitive impairment does not exert an influence on these scores as per the results observed in our study.

Kertzman et al. compared cognitive impairment with HAM-D scores in elderly patients and patients with dementia. The finding of these studies revealed no significant effect of cognitive impairment on HAM-D scores, which is similar to the observations of the current study [23]. Mulsant et al. assessed HAM-D scores in patients with cognitive impairment and physical illness and reported that cognitive impairment had no significant effect on HAM-D scores [24]. Schrag et al.'s study reported that HAM-D and MADRS scores could be considered valid for screening depression in patients with Parkinson's disease [25].

Though HAM-D and MADRS scores were not correlated with P300 in the present study, we recommend further studies with a larger sample size in patients with depression and also various simple tests to assess cognition. These tests should be done along with P300 [26].

Limitations

A single-centric study with small sample size is one of the major limiting factors. This needs to be followed up by more extensive cohort studies where patients with different profiles are assessed.

## Conclusions

In a nutshell, cognitive impairment as objectively observed on P300 ERP parameters did not have a significant association with the HAM-D scores and MADRS scores which are routinely used to assess the severity of depression in patients with newly diagnosed MDD. The statistical insignificance can be due to many confounding factors such as general level of functioning, motivation in the test situation, and quality of sleep prior to the test. Also, P300 studies necessitate a high level of concentration and attention. Many individuals did not understand the examiner's directions, which results in suboptimal waveform generation because it averaged out the waves over the entire recording session. However, our findings were contradictory to the findings of previously published studies which established a significant correlation between the two, we suggest more prospective studies with larger sample sizes to confirm the findings.
